# Women’s representation in higher leadership positions in Ethiopia in the last three decades since 1991

**DOI:** 10.12688/f1000research.150421.1

**Published:** 2024-07-02

**Authors:** Zeynie Chekol Degu, Flimon Hadaro Hando

**Affiliations:** 1 Development Studies, Addis Ababa University, Addis Ababa, Addis Ababa, 1176, Ethiopia

**Keywords:** Women’s representation, higher leadership positions, Gender equality

## Abstract

Gender equality in decision-making positions is crucial to achieving the goals of good governance, peace, democracy, and inclusive/sustainable development. The major aim of this research article is to investigate the representation trend of women in higher decision-making positions over the last thirty years, since 1991. The federal three organs of government (law formulating, law enforcing, and law interpreting bodies) were the focus of this research. This research is a mixed type of research that inculcates both qualitative and quantitative data types. Secondary data sources from relevant government institutions were mostly used. The data was analyzed through content analysis of documents and presented via descriptive data presenting techniques. The research findings reveal that although women’s representation in positions of decision-making has advanced considerably in recent years, the empirical data throughout the previous thirty years demonstrated the underrepresentation of women in higher leadership positions within the Ethiopian federal government. Furthermore, Women never held certain higher-level government leadership positions, such as the Prime Minister position, which seems to be forbidden for women. Women made up 23%, 19%, 19%, and 24% of the House of Peoples Representatives (HPR), the House of Federation (HoF), ministerial posts, and judicial bodies, respectively over the last three decades. Women are visibly underrepresented in the executive positions as compared to others. Thus, substantial policy and practical initiatives are needed to remove institutional, social, and economic barriers to boost women’s advanced visibility in senior leadership roles.

## 1. Introduction

### 1.1 Study background

The struggle for gender equality is a historical movement for equality, democracy, and women’s rights, and the interventions implemented for gender equality are important tools to enhance women’s enjoyment and participation in social, economic, and political decision-making areas ( Hearn & Husu, 2016). All levels of leadership positions in public institutions, civil society organizations (CSOs), non-governmental organizations (NGOs), and private organizations should be gender inclusive and take gender diversity into consideration, according to the findings of empirical literature ( Offermann & Foley, 2020). It is because, as argued by Ojulu and Melesse (2014); Ballington and Karam (2005), it is impossible to realize a participatory, genuine, accountable, and transparent governance system without the full and equal involvement of women. This also concerned by the convention of the Beijing platform for action that stated goals of peace and development and human rights could not be realized without incorporation of women’s perspectives at all levels of decision-making ( UN Women, 1995).

Even though, women make up half of world population and fifty percent of the working force, they are underrepresented in positions of leadership and decision-making across the globe ( Montecinos, 2017; Ballington & Karam, 2005). Around the world, women are primarily found in supporting roles; in junior and middle managerial roles ( Schedlitzki & Edwards, 2014). As a manifestation, at present women only constitute 16.1% of ministerial positions and only 22.9% of shares in parliaments at the global level, with the rest of the seats being held by men ( WEF, 2022, p. 39). According to the data released by UN Women in collaboration with Inter Parliamentary Union in “Women in politics: 2023” map, as of January 2023, solely 31 countries have head of states and head of governments. Out of the total 193 countries of the globe, only 17/151 countries (11.3%) have women head of states and 19/193 countries (9.8%) have women head of government. Given challenges are varied across countries according to different contexts, socio-economic barriers (resource shortage, illiteracy, multiple burdens, stereotype); psychological barriers (lack of confidence and motivation, perception to politics, lack of qualification); workplace challenges (lack of social-networks and sexual harassment); and political related challenges (male-friendly norms, ineffective women groups) are identified as major barriers that limit women from ascending to higher political leadership positions ( Tabassum, & Rafiq, 2023; Pranathi & Lathabhavan, 2021; Appelbaum et al., 2019 and Ballington & Karam, 2005).

In Ethiopian context, Tesfaye, Hirut, and Rahel (2019) noted that women have made significant contributions to Ethiopian history in the economic, social, and political affairs. However, according to Mihiret (2019) their strength, abilities, and power to do everything do not grant them the appropriate place, including formal authority for leadership. Facts and figures also demonstrated women’s lower participation in decision-making positions ( Negussie & Adula, 2021). For instance according to Negussie and Adula (2021) in Ethiopia since 1995 the first round national election, the involvement of women in the parliament was optimistically increasing but still not equal with men. As the data released by WEF (2022), in Ethiopia from the total seats in the upper house (House of Federation) only 30.60% of the seats are held by women (p. 165). Moreover, women are underrepresented in the Ethiopian justice system, both at the federal and regional levels ( Addadzi-Koom & Gage, 2022), In general, Ethiopia ranked 74th out of 146 countries in terms of gender gap parity ( WEF, 2022). According to the Inter-Parliamentary Union (IPU) (2017), institutionalization of democracy and producing inclusive public policy would not be realized without allowing the full and equal participation of women like that of men. Therefore, women and leadership-related research is required to investigate the issues of women in leadership in Ethiopia intensively to contribute some values on the efforts of making women’s perspective, interest and experience in the law making and enforcing process.

### 1.2 Empirical literature

Previous studies have been undertaken on women and leadership themes in Africa and Ethiopia too. In Africa, the 18-year state of knowledge production on African women in leadership and management has been examined by Nkomo and Ngambi (2009) in their article entitled ‘African Women in Leadership: Current Knowledge and a Framework for Future Studies’. The authors have done an extensive review of existing published research, including journal articles, books, book chapters, and monographs that have been published from 1990–2008 (18 years).

Among a total 43 publication reviewed by the authors, the largest body of knowledge has been produced on women’s leadership and management status is in case of South Africa, followed by Nigeria and Ghana. The findings of their review disclosed the underrepresentation of women in public office and political leadership in Africa. The majority of the empirical studies have focused on the factors that affect women’s performance in elevating them to higher leadership positions. Socialization, limited educational attainment, multiple roles, gender stereotyping, subtle discrimination, and organizational policies and procedures are the major factors identified by the studies. Indeed, there have been publications in Africa on women and leadership since 2010 by Bauer, Darkwah, and Patterson (2017), Ndlovu and Mutale (2013), Bauer and Okpotor (2013), and Amina and Ibrahim (2019). All these authors are optimistic about women’s growing participation in leadership positions on the African continent, as witnessed in Rwanda from East Africa and Algeria from Arab countries. As per the authors, the increase in women’s movements, quota systems, multi-party systems, educational opportunities, funding from international institutions, global and national agreements and commitments, and role models of women leaders, were found to be factors that contributed to raising women’s political participation in Africa.

There are numerous empirical studies that have been conducted on women and leadership in Ethiopia. However, a critical review of the existing literature found them as case studies conducted at banks, some selected government institutions, and woredas; furthermore, the concerns of writers are solely restricted to some specific aspects of women and leadership, such as challenges faced by women, women’s perceptions towards leadership, women’s leadership styles, and opportunities provided by institutions to empower women and related issues ( Worku, 2017; Tesfay, 2013; Shimelis, 2015; Bizualem & Kasaye, 2020; Mekasha, 2017; Endale, 2014; Genet, 2020; Gojjam and Singh, 2015; and Miressa, 2014).

Furthermore, there are few empirical studies that base themselves on the national context of women’s representation in political leadership positions by Meaza (2009); Berouk (2004); Ojulu and Melesse (2014). As it has been examined by the researcher of this study, all empirical studies have described the status of women’s representation in higher decision-making positions such as parliament, ministerial positions, and political parties since 1995 up until 2012, and they all disclosed the wider gender gap and insignificant representation of women’s in higher political leadership positions in Ethiopia. But, in the course of examining all these stated works, the researcher identified two basic gaps that needed to be filled by this study. The first is time; the above studies have covered the representation status of women in higher leadership positions from 1995–2012 E.C., or only in the four terms of elections. The second is the conceptual gap; the studies skipped the representation status of women in the third organ of government, which is the judiciary, and as per Bauer (2015), very little work has focused on women in judiciaries. Thus, this study intends to use the above studies as a springboard to frame the study and fill the mentioned gaps by expanding the time frame and conceptual coverage (scope).

Therefore, the purpose of this article is to address one of the objectives of the ongoing PhD dissertation on women’s representation in political leadership positions, and its central aim is to investigate the 30 years of women’s representation status at higher decision-making positions (federal-level three organs of government) in Ethiopia since 1991. This study mainly focused on the numerical representations of women and was not substantial.

### 1.3 Methods

This study employed a mixed-methods research approach by incorporating both qualitative and quantitative data types. The quantitative data provides a numerical and figurative description of women’s representation status in higher leadership positions over the past 30 years, while the qualitative data explores the implications of the raw data by referring to related empirical studies. This study primarily depends on secondary data sources. Records from higher government institutions such as the House of People’s Representatives, the House of Federation, and other pertinent institutions and documents provided the intended secondary data for the study. After obtaining data, a desk review of the most important findings and a content analysis of the documents were conducted. The data was presented in descriptive statistics such as percentages, tables, and figures.

This research article has approved by Addis Ababa University, College of development studies institutional review board named IRB-CoDS on April 3 ^rd^ 2024 under the written credential number 067/03/2024.

## 2. Findings and discussions

### 2.1 Women’s representation in the two houses (HoPR and HoF)

**2.1.1 Women’s representation in the Federal Legislature of Ethiopia post 1991**

As indicated in Table 1 below, Women’s representation in the Ethiopian national legislative (Parliament) varied throughout the past thirty years. Despite a recent notable increase, women’s participation in the legislature has been slowly increasing since the first round of national elections in 1995 and the establishment of the federal state structure and parliamentary system of government. For instance, when the House of people representatives first assembled in 1995, its membership consisted of 97% men and 2% women. This is to mean that there were no female representatives to shape policy or voice their experiences, opinions, or interests on a variety of issues.

**Table 1.** Female representation in the House of Peoples Representatives (HoPR) (1995 – 2027).

Rol. No.	Election terms (Years)	Total seats	Male MP	Female MP	Male speakers	Female speakers	Male vice-speakers	Female vice-speakers
1	1995-2000	547	533 (97%)	14 (2.6%)	1	0	1	0
2	2001-2005	547	505 (92%)	42 (8%)	1	0	0	1
3	2006-2010	547	430 (79%)	117 (21%)	1	0	0	1
4	2011-2015	547	395 (72%)	152 (28%)	1	0	0	1
5	2016-2021	547	334 (61%)	213 (39%)	0	1	0	1
6	2022-2027	472	275 (58%)	197 (42%)	1	0	0	1
Total Part.	1995-2027		77%	23%	83%	17%	17%	83%

**Source**: FDRE, HoPR Teaching & Communication Directorate.

From the second to the sixth round of national elections, women’s representation continued at a gradual but promising pace, with 8%, 21%, 28%, 39%, and 42% of parliamentary seats being held by female legislators, respectively. According to the study’s findings, there has been a 6.6% rise in the average representation of women in the HPR through the previous 30 years. In addition, women have only made up 23% of the seats in the Ethiopian national parliament on average over the past thirty years, with men holding the remaining 77% of the seats. In addition to parliament membership, speaker and deputy speaker positions are among the other higher decision-making positions in the Ethiopian legislature. Women had a higher representation in the deputy speaker roles throughout these election terms; in reality, during the last 30 years in the HoPR, there has only been one female speaker. Men tend to occupy speaker positions more frequently.

Overall, this study’s findings show that, despite women’s representation growing over time, men have typically constituted an excessive number of legislators in Ethiopia. This conclusion also confirmed the study results published in 2009 by Meaza Ashenafi, 2004 by Berouk Mesfin, and 2014 by Ojulu and Melesse, which indicated that women’s presence in senior political decision-making positions in Ethiopia is insignificant.

This research finding implies that the problems, interests, ideas, values, and roles of women have been overlooked by the legislature because of their underrepresentation. According to Oyindamola and Olaniyan (2020), men have little concern and attention for women’s issues as a result of gendered socialization. In line with this, Shimelis (2015) raised the experience and interest arguments, and according to these arguments, women’s experiences, perspectives, and interests are different from those of men’s, which influence policy decisions in different ways, so representative institutions are essential to articulating the concerns of women’s. The other implications of this finding are that half of the population of the country is not well represented in parliament, given that women constitute over 50% of the nation’s total population.

On the other hand, as Oyindamola and Olaniyan (2020) discovered, fewer women in the legislature entails less attention to social policy (human trafficking, issues of children, youth, and seniors), gender equality, and family policy. An empirical study conducted by Devlin and Elgie (2008) and O’Brien and Piscopo (2019) also gives support to this argument, acknowledging that a higher representation of women in parliament has a significant impact on the legislative agenda and environment (women-friendly working hours and calendar). Finally, as argued by Ballington and Karam (2005), the insignificant representation of women in parliament is a manifestation of poor democracy. A democratic institution devoid of women’s full participation is retrograde, and any country that upholds it cannot advance politically or flourish economically ( Oyindamola & Olaniyan, 2020).

**2.1.2 Women’s representation in the Standing Committee Chairs in HoPR since 1991**

The information presented in Table 2 attached shows how the number and purview of standing committees have changed over time in response to Ethiopia’s changing political and socioeconomic landscape. As a result, the number of standing committees varies throughout time. The largest standing committees (20) were formed in 2018 on a variety of areas; however, when a new structure (the creation of multiple subcommittees on several grand committees) was implemented in 2019, the number of standing committees dropped to 10.

**Table 2.** Female representation in HoPR Standing Committee Chairs (1995 – 2022).

Rol. No.	Service years	Number of standing committees	Chairpersons		Vice chairpersons		Remarks
			Male (%)	Female (%)	Male (%)	Female (%)	
1	1996-2000	9	8 (89)	1 (11)	8 (89)	1 (11)	Various Sub-committees have been formed in 2019 and there were some female chairs in the committees.
2	2001-2005	12	11 (92)	1 (8)	11 (92)	1 (8)	
3	2006-2010	13	10 (76)	3 (23)	9 (69)	4 (31)	
4	2011-2015	16	12 (75)	4 (25)	11 (69)	5 (31)	
5	2016-2017	18	11 (61)	7 (39)	8 (44)	10 (56)	
6	2018-2019	20	12 (60)	8 (40)	10 (50)	10 (50)	
7	2019-2021	10	4 (40)	6 (60)	8 (80)	2 (20)	
8	2022	11	6 (55)	5 (45)	7 (64)	4 (36)	
**Total Chairs**			68.5%	31.5%	70%	30%	

**Source**: FDRE, HoPR Teaching & Communication Directorate.

As can be seen in the table, men significantly outnumber women in Ethiopian parliament standing committee chair and deputy chair posts held after 1991. To put it numerically, in the past 30 years, the proportion of women as chairperson in the HPR standing committees has merely by 31.5%. The participation of women as vice chairpersons is lower than this number, at 30%. Unlike the HoPR deputy chairperson positions (the position that seems left for female representatives), even this position in the standing committees is highly represented by men’s. As a result, throughout the past three decades, the proportion of women serving as chairpersons and deputy chairpersons has only been 30.5%; male members of parliament have held the remaining 70% of these positions. Thus, women have a small presence among the chairs of the standing committees in the HoPR, much like they have little representation among parliamentarians.

According to Benda (1997), given differences in their type, duties, and significance, most countries in the world adopted parliament (standing) committees that specialize on various matters, and they are usually created for the effectiveness and efficiency of the large work of the legislature. Parliamentary chairs are considered important policy actors, and they play an important role in parliamentary political leadership positions ( Gaines et al., 2019). A legislature with strong committees and chairs has an impact on shaping government policies. According to Fortunato et al. (2019), committee chairs can play two important roles by using their agenda powers: encouraging opposition political parties to examine proposed government policies and enabling the ruling parties to provide better policies to the public. To sum up, women chairs are essential to the advancement of gender equality, inclusive governance, and the development of policies that are advantageous to society at large because of their concern for grassroots agendas ( Wängnerud, 2009).

Therefore, the underrepresentation of women in committee chairs and other political leadership roles inside the parliament has implications of its own. The first is the absence of female role models, which would deter women from entering the political arena and positions of leadership. Secondly, inclusive decision-making would be missing. Their limited presence in political leadership roles within parliament undermines inclusive decision-making procedures that welcome a range of viewpoints and opinions. The third implication is that there would not be policy prioritization, i.e., women are more likely to focus on policies that address women’s rights, gender equality, social welfare education, and other related issues.

**2.1.3 Women’s representation trend in the House of Federation (HoF) or Upper House post 1991**

The data presented in Table 3 attached below deals with women’s representation in Ethiopia’s House of Federation, the country’s second chamber of parliament. In the past thirty years, Ethiopia has held six consecutive national elections. Only seven women were elected to the house of federation during the first national election in 1995, with 103 seats being held by men, according to the facts shown by figures. Over the last six terms, unlike the case of House of Peoples Representatives, the highest number of female representations in the upper house (HoF) registered in the 2015 national elections, constituting 50 (32.6%) of seats. The number of female representatives in this house did not exceed 50 of the total members. However, this number was again reduced to 43 (30%) in the recent 2021 national election due to the absence of Tigray regional state representatives due to the official war between the federal government and the TPLF (Tigray People’s Liberation Front), which started in November 2020 and ended in November 2022.

**Table 3.** Females representation in HoF over the six terms of elections (1995 – 2027).

Numbers	Terms of election	Male members	Female members	Total
1	1995	103 (93.3%)	7 (6.7%)	110
2	2000	104 (92.9%)	8 (7.1%)	112
3	2005	98 (81.7%)	22 (18.3%)	120
4	2010	111 (82.3%)	24 (17.7%)	135
5	2015	103 (67.4%)	50 (32.6%)	153
6	2021	101 (70%)	43 (30%)	144
**Total women’s representation over the six terms**	**1995-2027**	**81 %**	**19%**	**774(100%)**

**Source:** House of Federation Women and Children Affair’s Office.

According to the results of this study, males and females were represented in the House of Federation by 81% and 19%, respectively, over the last thirty years (six terms). This shows that, on average, women’s were underrepresented in the House of Federation compared to even the representation level of women’s in the House of Peoples Representatives. The findings of this study further revealed that, despite the dynamics of the number of female representatives at different terms, female representation in the upper house has increased by 3.88% over the course of the past six terms. Generally, given their underrepresentation in both houses, the above data discloses that women are insignificantly represented in the House of Federation compared to the House of People’s representatives.

Meanwhile, Women make up a minor portion of the House’s standing committees in addition to being members of the House of Federation. Data from 2010 to the most recent election (2021) showed that, on average, women’s participation in standing committees was only 17% throughout the previous three terms. Women are also disproportionately underrepresented in the working four standing committees, with the standing committees on revenue affairs, budget, and subsidies having the lowest representation, followed by the council of constitutional inquiry.

This finding supports the results released by Atsede, Aemro, and Eyayu (2022), who argued that despite attitudinal changes and governmental efforts regarding women’s political participation enhancements, “nevertheless, facts and figures show that, even currently, the participation of women is not at an equal level with men in the highest decision-making spheres” (p. 75). And this finding implies that without a fair and equitable representation of women in the political process, the objectives of democracy, good governance, human rights, gender equality, and development would not be realized. It is because, as argued by Wubante (2021), the involvement of women in political leadership is essential for promoting democracy and establishing effective governance. On the other hand, women’s lower engagement in the HoF means the absence of women’s voice during constitutional interpretation, constitutional amendments, and on the issues of rights of nations, nationalities, and peoples of Ethiopia, which has an adverse effect on the impacts of women’s participation in national and regional agendas.

### 2.2 Women’s representation in the Law Enforcing (Executive) Body

**2.2.1 Women representation in Chief Executive and President Positions**

National executive positions including head of government are the highest political leadership positions of a state in which important national-based decisions are undertaken and are responsible for the supervision and implementation of policies and laws made by the legislature. In the Ethiopian context, these positions are mostly occupied by male executives such as male prime ministers, male presidents, and male deputy prime ministers. As per the figures in Table 4 pointed out, shockingly, there was no female prime minister and there were no female presidents (until President Sahle-Work Zewde was elected as the first female president in 2018) in Ethiopian history post-1991. Meanwhile, only one female deputy prime minister served in the position of 2 ^nd^ deputy prime minister from 2014–2016, while all deputy prime minister positions were held by male executives after 1991. And her tenure was not longer than two years.

**Table 4.** Female representation in Chief Executive and President Positions (1991 – 2024).

Roll. No.	Term of office	Prime Ministers		Deputy Prime ministers		Presidents	
		Male	Female	Male	Female	Male	Female
1	1991-1995	1	0	1	0	1	0
2	1995-2012	1	0	1	0	1	0
3	2012-2018	1	0	1	1	1	0
4	2018-Present	1	0	1	0	0	1

**Source**: FDRE, HoPR Teaching & Communication Directorate.

The findings of this study revealed that women have been severely underrepresented in Ethiopian executive posts since 1991, which contradicts the findings of Farida Jalalzai’s (2004) global-based work, in which she concluded that women have rarely been presidents or prime ministers around the world; however, more women have been reaching these high positions since the 1990s. Her findings are invalid in the context of Ethiopia. On the other hand, the findings of this study contradict her other finding, which claimed that women are more likely to access executive positions in the parliamentary government system than in the presidential system (the argument having that women are less likely to be elected directly by the public due to patriarchal society) ( Jalalzai, 2004). Her finding appears to be unsound, given that Ethiopia’s parliamentary system experience over the last thirty years has not provided opportunities for women to advance to such top decision-making positions.

According to the 1995 FDRE constitution (Article 72/1), the Prime Minister and the Council of Ministers have the highest executive authorities of the Federal Government of Ethiopia, and the Prime Minister in particular has crucial national powers. This implies that women’s absence as prime minister since 1991 means that over the past 30 years, women’s did not organize cabinet ministers (councils of ministers) for different departments; women’s never been the commander-in-chief of the national armed forces (all past 30 years, defense force movements and war-related decisions have been done by males); posts of commissioners; the president and vice president of the federal supreme court; and the auditor general have never been selected by females. Thus, the existing economic condition, justice system, diplomacy, and peace trend are or were the result of men’s political leadership. This implies that this and related conditions could have been different in the country if male leaders collaborated with females and considered gender inclusivity in leadership positions.

**2.2.2 Post 1991 Women’s representation at the Ministerial Positions and Their Portfolios**

Government ministries are mainly responsible for the implementation of laws and regulations and for the administrative functions of the national government. As it is observed in Table 5 attached below, through the course of the last 30 years, in Ethiopia, the number and nomenclature of ministerial offices have been changing to cope up with the socio-economic, political, and technological changes of different times.

**Table 5.** Female representation in Ministerial Positions and their Portfolio (1995 – 2022).

Ro. No.	Term of office	No. of ministerial offices	Female ministers	Male ministers
1	1995-2001	17	1 (5.8%)	16 (94.2%)
2	2002-2005	18	1 (5.5%)	17 (94.5%)
3	2006-2008	20	2 (10%)	18 (90%)
4	2009-2010	21	1 (4.76%)	20 (95.24%)
5	2011-2012	23	3 (13%)	20 (87%)
6	2013-2015	23	5 (22%)	18 (78%)
7	2016-2018	30	8 (27%)	22 (73%)
8	2019-2021	21	10 (48%)	11 (52%)
9	2022	22	8 (36%)	14 (64%)
**Total in 30 years**			19%	81%

**Source**: FDRE, HoPR Teaching & Communication Directorate.

Throughout this journey, the biggest number of female ministers was three (13%) until 2013, while male ministers occupied 87% of ministerial positions. Since 2013, the proportion of female ministers has increased to five (22%) or more. The highest number of female ministers were appointed in 2019, when the percentage of them increased to 10 (48%), or nearly half of the total share. This time, the male/female minister ratio was 10 (48%)-11 (52%), however, the number of female ministers in ministerial positions, was recently lowered to 8 (36%), in 2022. According to the study’s findings, female representation in ministerial positions has averaged 19% during the last thirty years. Furthermore, the average female’s leadership in ministerial positions increased gradually by 3.44%.

This research comes up with the finding that, though currently the engagement of females at the ministerial posts is increasing and encouraging, there was no meaningful representation of women’s before 2019. On the other hand, according to the data obtained from the FDRE House of Peoples Representatives teaching and communication directorate, women in Ethiopia have been appointed in every ministerial office at least once over the last thirty years, but the top three departments (ministerial positions) that are frequently headed by female ministers are the ministries of women, children, youth, and social affairs (over eleven times), culture and tourism (five times), and urban development and construction (four times). The results of this study showed that, despite the fact that these ministries contribute to the socio-economic and political advancement of a nation, women in Ethiopia have not held the leadership positions in important and high-profile political posts, such as those related to science and technology, foreign affairs, finance, defense, and justice. According to Barnes & Taylor-Robinson (2018), these positions allow ministers to represent their nation at significant global forums, and prime ministers and presidents frequently use them to express the general direction of their government’s policies. As concluded by Krook and O’Brien (2012), women have held fewer cabinet positions, and when they did, they were frequently assigned to portfolios associated with lower status and “feminine” attributes. The findings of this study also confirm the findings revealed by Barnes & Taylor-Robinson (2018); although the importance of cabinet portfolios varies across countries, the most common high-profile posts are defense, finance, and foreign relations. Even though these authors underlined the importance of women’s presence in powerful positions as, “women’s presence in top cabinet posts is positively associated with both women’s and men’s satisfaction with and confidence in government” ( Barnes & Taylor-Robinson, 2018, p.19). However, it is not common for women to hold these important positions; in particular, certain leaders in Asia, the Middle East, and Sub-Saharan Africa have never proposed a woman to take any of these positions ( Barnes & Taylor-Robinson, 2018).

### 2.3 Women’s representation in the Federal Law Interpreting Body

**2.3.1 Women’s representation trend in the Federal Courts since 1991**

Figures in Table 6 attached below reveal the share of males and females as judges and in the positions of president and vice president in the three levels of federal courts, such as the Federal First Instance Courts (FFICs), Federal High Courts (FHCs), and the Federal Supreme Court (FSC). As per the data in Table 6, the federal government has appointed a total of seven presidents in the three federal courts over the last thirty years. Among the seven presidents, only one was female, while six of the total presidents were male. According to the finding, Meaza Ashenafi was not only the first female president of federal courts but also the only female chief justice (president of the Federal Supreme Court) in Ethiopian history. Besides this, over the course of this year, a total of 11 vice presidents have been appointed, and among them, 9 were males and the rest 2 were females. Within these years, the representation of female leaders in the federal courts president and vice president posts was 14% and 25%, respectively.

**Table 6.** Female representation in the Federal Courts (1996 – 2022).

Years	Federal courts	Presidents		Vice-presidents		Number of appointed judges		Total number of appointed judges
		Male	Female	Male	Female	Male	Female	
1996-2001	Federal Supreme Court	1	0	1	0	12 (75%)	4 (25%)	16
2002-2008		_	_	_	_	9 (90%)	1 (10%)	10
2009-2015		1	0	1	0	17 (94%)	1 (6%)	18
2016-2021		0	1	1	0	21 (66%)	11 (34%)	32
1996-2001	Federal Highest Court	1	0	1	0	48 (87%)	7 (13%)	55
2002-2008		_	_	1	0	38 (88%)	5 (12%)	43
2009-2015		_	_	_	_	39 (80%)	10 (20%)	49
2016-2021		1	0	1	1	87 (72%)	34 (28%)	121
1996-2001	Federal First Instance Court	1	0	_	_	88 (83%)	18 (17%)	106
2002-2008		_	_	1	0	40 (69%)	18 (31%)	58
2009-2015		_	_	_	_	70 (71%)	28 (29%)	98
2016-2021		1	0	2	1	139 (70%)	60 (30%)	199
Total judges	Three courts	6 (86%)	1(14%)	75%	2 (25%)	608 (76%)	197 (24%)	805 =100%

**Source:** FDRE, HoPR Teaching & Communication Directorate.

On the other hand, across the last 30 years since 1991, a total of 805 judges have been appointed to the federal courts, and among them, the proportion of female judges was 197 (24%), while the number of male judges was 608 (76%). Meanwhile, independently, the share of female judges in the Federal First Instance Courts (FFICs), Federal High Courts (FHCs), and the Federal Supreme Court (FSC) across the last thirty years was 27%, 21%, and 22.3%, respectively. From this, it is sound to deduce that the representations of female judges are better at the federal first instance court than the other two federal courts.

Generally, for the past thirty years, there have only been 24% of women serving as federal judges, and only 14% and 25% of them have held leadership roles as president and vice president, respectively. The study’s main conclusion was that women are underrepresented in Ethiopia’s legal system, both in leadership roles and in arbitration. This finding is consistent with the conclusion of Addadzi-Koom and Gage (2022), which announced that in the three levels of Ethiopian federal courts, the total number of female judges is outnumbered by male judges. This finding is also consistent with the global-based empirical research by Castillejos-Aragón (2021), which affirmed that despite the growing participation of women in the legal profession, the gender gap remains prominent, and according to this writer, women’s are still underrepresented in top-ranking positions of the judiciary, even in countries where more women are appointed as judges.

As pointed out by different writers, the lower representation and participation of women in the judiciary body have their own implications. As argued by Castillejos-Aragón (2021), women’s absence in the judiciary reduces the responsiveness, inclusivity, and participatory nature of decision-making at all levels. It also has an impact on women’s equal visibility, diversity of viewpoints, and gender-sensitive judicial institutions. Additionally, the legitimacy of the judiciary would be reduced by the lack of women in this body and last but not least it neglects women’s inherent right, acknowledged by various international frameworks, to participate equally in all public institutions ( Castillejos-Aragón, 2021).

## 3. Conclusion

In Ethiopia, women’s representation in senior leadership roles within the federal government was and remains insignificant. These roles include head of state and government, cabinet positions, membership in the House of People’s Representatives and House of Federation, chairs of standing committees, and the judiciary body. Women do not hold certain higher-level decision-making roles, such as the Prime Minister position, which seems to be closed to women.

The percentage of women serving in Ethiopia’s national legislature, or Parliament, has increased since 1991. The study’s findings indicate that the proportion of women in the HoPR has increased by an average of 6.6% over the preceding thirty years. Furthermore, throughout the preceding 30 years, women’s representation in the Ethiopian national parliament has averaged only 23%. In the meantime, women have a stronger representation in the deputy speaker roles during this election term. Speaker roles are typically occupied by men. Overall, this study’s findings show that, despite women’s representation growing over time, men have consistently constituted an excessive number of legislators in Ethiopia. According to this study, female membership in the HoF constituted solely 19% in the last three decades. This demonstrates that women’s presence in the House of Federation is negligible when compared to the representatives in the House of People, considering their underrepresentation in both houses.

High executive positions in Ethiopia are typically held by men’s, including those of presidents, deputy prime ministers, and prime ministers. Amazingly, throughout Ethiopian history since 1991, there has never been a female prime minister or president (before to President Sahle-work Zewde’s election as the country’s first female president in 2018). Over the past thirty years, the average percentage of women holding ministerial positions has been 19%. On average, there has been a modest increase of 3.44% in the number of women holding ministerial positions. The ministry of women, children, youth, and social affairs; the ministry of culture and tourism; and the ministry of urban development and construction are the top three departments (ministerial roles) that are frequently headed by female ministers, according to this study.

The study’s primary finding also demonstrated the minimal representation of women in leadership roles and arbitration within the Ethiopian legal system. During the entire year, the judiciary organ had 14% and 25% of female leaders in the positions of president and vice president of the federal courts, respectively. Furthermore, of the total 805 judges, the proportion of female judges was solely 197 (24%).

All in all, the data and statistics presented in this study point to a hopeful increase in the representation of women in positions of decision-making. For women to effectively participate, however, substantial policy and practical initiatives including appropriate monitoring and evaluation are needed to remove institutional, social, and economic barriers as well as increase women’s advanced visibility in senior leadership roles.

Specifically, to enhance women’s participation in higher leadership positions, policies that are inclusive, strong, and have important indicators and measurements on women’s civil rights, women’s participation in civil society, and women’s engagement in decision-making positions need to be developed in the meantime, particularly taking into account the gaps in Ethiopia’s current policy frameworks.

### Ethics and consent

All the relevant sources reviewed are included in the references and citations. No source (data analyzed and scholarly works reviewed) is used without proper acknowledgement. This research article has approved by Addis Ababa University, College of development studies institutional review board named IRB-CoDS on April 3 ^rd^ 2024 under the written credential number (ethical approval number) 067/03/2024. Data was collected from relevant government institutions, not from individual participants. Since all of what is contained in the article was acquired through document analysis, verbal approval was reached with the appropriate body for the publication of the secondary data obtained from them. Furthermore, the university’s ethical approval committee assessed the research article’s ethical status and agreed to proceed with its publishing by granting an ethical approval certificate.

## Data Availability

Dryad: Women’s status in Higher leadership positions in Ethiopia since 1991 [Dataset], Dryad. Accessible via this link: https://doi.org/10.5061/dryad.7m0cfxq38 ( Chekol & Hadaro, 1991). The project contains the following underlying data and tables: Women_in_ legislative_body; Women_in_the_executive_body; Women_in_the_judiciary_body and Women_in_the_upper house_ (HoF). It has also included the extended data file; guiding_questions_for_secondary_data The data is available for peer review under the link: https://datadryad.org/stash/share/YAFozIzc81uzmSfYy_jjLbgdmrpodbQm6j1UOUyemSg. Data are available under the terms of a CC0 1.0 Universal (CC0 1.0) Public Domain Dedication license.
