# Supplementary material for: Women’s representation in higher leadership positions in Ethiopia in the last three decades since 1991
Source: F1000Res. 2024 Dec 6;13:729. Originally published 2024 Jul 2. [Version 2] doi: 10.12688/f1000research.150421.2 (PMC11273078; doi:10.12688/f1000research.150421.2)
Supplement: Peer review history

# Reviewer report: Reviewer response for version 2

**DOI:** 10.5256/f1000research.175654.r346589

**Published:** 2024-12-11

**Authors:** Professor Claes Maria Theresia

**Affiliations:** 1 Institute for Gender and Diversity in Organization, Vienna, Austria

This article is acceptable for indexing.

Is the case presented with sufficient detail to be useful for teaching or other practitioners?

Yes

Is the work clearly and accurately presented and does it cite the current literature?

Yes

If applicable, is the statistical analysis and its interpretation appropriate?

Yes

Are all the source data underlying the results available to ensure full reproducibility?

Yes

Are the conclusions drawn adequately supported by the results?

Yes

Is the background of the case’s history and progression described in sufficient detail?

Yes

Reviewer Expertise:

NA

I confirm that I have read this submission and believe that I have an appropriate level of expertise to confirm that it is of an acceptable scientific standard.

# Reviewer report: Reviewer response for version 1

**DOI:** 10.5256/f1000research.164993.r299158

**Published:** 2024-07-25

**Authors:** Jiregna Tadesse

**Affiliations:** 1 Alice Salomon University of Applied science, Berlin, Germany

**Constructive Assessment of the Study**

 The case study on women’s political representation in Ethiopian leadership positions is well-structured and provides a comprehensive examination of the subject. Below is a detailed assessment along with answers to the mandatory reviewer questions and recommendations for potential improvements.

**Background and Historical Context**

**Assessment:**

 The study offers a thorough historical and contextual overview of women's involvement in Ethiopian politics. It effectively outlines the evolution of gender representation and key milestones affecting women’s political participation.

**Recommendations:**

 To further enhance the background, consider incorporating a detailed timeline of significant legislative and societal changes that have impacted women’s roles in politics. This would provide a clearer understanding of the historical progression and its effects on the current political landscape.

**Clarity and Accuracy of Presentation**

**Assessment:**

 The presentation of the study is clear and well-organized, with accurate references to the current literature. It aligns with existing research and contributes meaningfully to the discourse on gender and leadership.

**Recommendations:**

 Continue to ensure clarity throughout the study, particularly when presenting complex data or historical developments. Double-check all citations for accuracy and completeness to maintain the study's scholarly integrity.

**Statistical Analysis and Interpretation**

**Assessment:**

 The study employs appropriate statistical methods, and the interpretation of results is consistent with the study’s objectives. This adds robustness to the findings and supports a better understanding of women’s representation in Ethiopian politics.

**Recommendations:**

 Ensure that the methodologies used are described in detail, including any assumptions and potential biases. Clear and transparent presentation of statistical results will further validate the study's conclusions.

**Reproducibility of Source Data**

**Assessment:**

 The availability of source data is essential for verifying the study’s findings. While the study’s summary does not specify data accessibility, ensuring that underlying data is available will support reproducibility and transparency.

**Recommendations:**

 Make all data sources accessible and consider including supplementary materials or appendices that outline the data collection process. This will enhance the reproducibility of the study and provide greater transparency.

**Support for Conclusions**

**Assessment:**

 The conclusions drawn from the study are well-supported by the results presented. The study successfully connects its findings to broader themes in gender representation and political participation.

**Recommendations:**

 Review the conclusions to ensure they are directly supported by the data and analysis. Address any limitations in the study’s results and discuss their potential impact on the conclusions.

**Detail and Usefulness for Teaching or Practitioners**

**Assessment:**

 The study is presented with sufficient detail to be valuable for both teaching and practical applications. It provides meaningful insights into the challenges and advancements in women’s political leadership.

**Recommendations:**

 Enhance the practical relevance of the study by including specific case studies or examples that illustrate key findings. This will increase its utility for practitioners and educators in understanding and addressing issues related to women’s leadership.

**Mandatory Reviewer Questions**

 1. Is the background of the case’s history and progression described in sufficient detail?

    Yes, the background is described in sufficient detail, providing a thorough historical and contextual overview of women’s political involvement in Ethiopia. The study effectively captures the evolution and key milestones in women’s representation.

 2. Is the work clearly and accurately presented and does it cite the current literature?

    Yes, the study is clearly and accurately presented. It appropriately cites current literature, aligning with existing research and contributing meaningfully to the field of gender and leadership.

 3. If applicable, is the statistical analysis and its interpretation appropriate?

    Yes, the statistical analysis is appropriate, and the interpretation of results is consistent with the study’s objectives. The analysis adds robustness to the findings and enhances understanding of women’s representation.

 4. Are all the source data underlying the results available to ensure full reproducibility?

    Not fully specified. The summary does not mention the availability of source data. To ensure reproducibility, it is recommended to make all source data accessible and consider including supplementary materials or appendices detailing the data collection process.

 5. Are the conclusions drawn adequately supported by the results?

    Yes, the conclusions are well-supported by the results. The study effectively connects its findings to broader themes in gender representation and political participation, though it is advisable to review and address any limitations that may impact the conclusions.

 6. Is the case presented with sufficient detail to be useful for teaching or other practitioners?

    Yes, the case is presented with sufficient detail to be valuable for teaching and practical applications. The study provides insights into challenges and advancements in women’s political leadership, though including specific case studies or examples could further enhance its practical relevance.

**Approval Status**

**Approved:** The study is well-executed, provides significant insights into women’s political representation in Ethiopia, and contributes meaningfully to existing literature. The recommendations provided aim to further strengthen the study and ensure its impact and applicability.

Is the case presented with sufficient detail to be useful for teaching or other practitioners?

Yes

Is the work clearly and accurately presented and does it cite the current literature?

Yes

If applicable, is the statistical analysis and its interpretation appropriate?

Yes

Are all the source data underlying the results available to ensure full reproducibility?

Yes

Are the conclusions drawn adequately supported by the results?

Yes

Is the background of the case’s history and progression described in sufficient detail?

Yes

Reviewer Expertise:

I am interested in exploring a research areas such as Sustainable Peace, Conflict Management, Conflict Transformation, Political Reform, National Dialogue and Consensus, Reconciliation, Migration and Displacement, Famine, War Studies, Humanitarian Governance, Humanitarian Business, Local Development, Sustainable Development, Environmental Governance, Transboundary Water Governance, Intersectional Approach, Human Rights, Racism, Discrimination, Gender Study, Political Economy of War, Armed Conflicts and Military Science, Post-Conflict State Reconstruction, Aid, and Society, Famine, and War Studies, Web Design, UI/UX, Artificial Intelligence, Machine Learning, Cyber Security and more.

I confirm that I have read this submission and believe that I have an appropriate level of expertise to confirm that it is of an acceptable scientific standard.

# Response

**Published:** 2024-07-31

**Authors:** Zeynie Chekol

**Affiliations:** Center for regional and local, Addis Ababa University, Addis Ababa, Addis Ababa, Ethiopia

Dear Dr. Jiregna Tadesse,

 Thank you very much for taking the time and employing your efforts to review our article, and I appreciate your commitment. All the comments and suggestions are greatly constructive and appreciated, and we will consider them in upgrading the manuscript to contribute to better scientific knowledge production in the theme of women and leadership.

 Thanks, again.

# Reviewer report: Reviewer response for version 1

**DOI:** 10.5256/f1000research.164993.r303834

**Published:** 2024-07-22

**Authors:** Professor Claes Maria Theresia

**Affiliations:** 1 Institute for Gender and Diversity in Organization, Vienna, Austria

This is an interesting article on Women’s representation in higher leadership positions in Ethiopia in the last three decades since 1991. It shows how women have progressed in political and judicial high positions in the last 30 years, but also that the numbers are still small. Another conclusion is that women do advance, but mostly into positions of lower responsibility and power, not in the higher or more powerful positions. Progress is small, with the result that political decisions are made by men, without necessarily taking into consideration half the population, or having their perspectives on the decisions.

 The text is well written, but needs to go trough language checker for minor typos and grammatically incorrect sentences.

 One remark also about the description of the article: the authors mention qualitative and quantitative research, but apparently the only method used is quantitative.

 The conclusion is well written and informative.

Is the case presented with sufficient detail to be useful for teaching or other practitioners?

Yes

Is the work clearly and accurately presented and does it cite the current literature?

Yes

If applicable, is the statistical analysis and its interpretation appropriate?

Yes

Are all the source data underlying the results available to ensure full reproducibility?

Yes

Are the conclusions drawn adequately supported by the results?

Yes

Is the background of the case’s history and progression described in sufficient detail?

Yes

Reviewer Expertise:

Gender and Diversity in Organisations, Leadership

I confirm that I have read this submission and believe that I have an appropriate level of expertise to confirm that it is of an acceptable scientific standard.

# Response

**Published:** 2024-07-22

**Authors:** Zeynie Chekol

**Affiliations:** Center for regional and local, Addis Ababa University, Addis Ababa, Addis Ababa, Ethiopia

Hello, dear Professor Claes Maria Theresia,

 Thank you for your commitment, time, and efforts to review our article, and I appreciate your constrictive and valuable comments. We will address all the reservations and improve them in a way to contribute to the scientific knowledge production on the theme of women and leadership. 

 Thanks again,

 Regards.
